# Stakeholder Perspectives on the School Food Environment: Insights from South African Learners—A Pilot Study

**DOI:** 10.3390/nu16203542

**Published:** 2024-10-18

**Authors:** Imana Pal, Ashika Naicker, Gilbert Tshitaudzi, Evonne Shanita Singh, Heleen Grobbelaar, Nokuthula Vilakazi

**Affiliations:** 1 Department of Food and Nutrition Consumer Sciences, Durban University of Technology, Durban 4001, South Africa; 2 UNICEF, Pretoria 0011, South Africa; gtshitaudzi@unicef.org

**Keywords:** stakeholder, school food environment, barriers, enablers, school food programme

## Abstract

Leveraging learner-driven insights to enhance the SFE can significantly influence food choices by decreasing the consumption of unhealthy foods and increasing access to healthier options. Using learners’ voices as important stakeholders in the school food environment (SFE), this qualitative research study aimed to gain a deeper understanding of their perspectives, identify barriers and enablers to fostering a healthy SFE in South African public schools, and explore the link between the SFE and learners’ food choices. Six focus group discussions (FGDs) with 4–6 participants were held in six schools, including three primary and three secondary schools in the KwaZulu-Natal Department of Education’s iLembe district, SA, with learners aged 12–14 and 15–18 years, respectively. Learners were asked how schools could help them eat healthier and how their SFE benefitted them. The data were analysed using thematic data analysis methods. The FGDs generated 14 themes and subthemes. Emerging information suggests a lack of control over the sale of unhealthy food despite the existence of guidelines. This is further exacerbated by the marketing of unhealthy foods, which prevents learners from selecting healthier options. Furthermore, they stated that promoting the school food programme (SFP) and changing attitudes towards healthy food intake through comprehensive nutrition education are useful ways to enhance the SFE. The findings of this study underscore the critical role of learner insights in shaping effective strategies to improve the SFE.

## 1. Introduction

The United Nations (UN) has designated the period from 2016 to 2025 as the Decade of Action on Nutrition. The goal is to support government policies and initiatives to eradicate all types of malnutrition, including undernutrition, vitamin and mineral deficiencies, and overweightness and obesity. Poor dietary choices are becoming a more significant contributor to obesity and non-communicable diseases (NCDs) connected to food on a worldwide scale [1].

In South Africa, childhood overweightness and obesity pose serious health and economic implications for individuals, families, and society at large [2]. According to the 2022 Global Nutrition Report [3], in 2016, the prevalence of overweightness and obesity in South Africa was 29.4% and 6.5% among girls and 20.2% and 9.8% among boys, respectively. Additionally, the issue of micronutrient deficiency (such as anaemia and vitamin A deficiency) has significant public health implications for the country. It is alarming that South Africa has a high worldwide prevalence of overweightness and obesity and ranks among the 34 countries with the worst childhood stunting [4]. This suggests that the country is facing a significant problem of the double burden of malnutrition.

The foundation for life and health is established in the first 8000 days of life [5]. Research suggests that dietary habits imprinted in these early years often persist into adulthood, underscoring the critical importance of understanding and shaping children’s dietary behaviours early on [6]. Much research has focused on individual cognitive factors as predictors of eating habits. Over the past decade, environmental factors have also been recognised for influencing health behaviours. This recognition has accelerated the use of ecological models to study health behaviours, including dietary habits [7]. These theories indicate that both cognitive processes and environmental factors may impact behaviour.

A comprehensive understanding of the food choices and accessibility challenges in South Africa’s school food environment (SFE) requires an understanding of the cultural information and specific information of the available variety of food. South Africa is a nation characterised by a rich cultural diversity, where different ethnic and socioeconomic groups have unique food habits. For instance, commonly consumed traditional meals such as maize porridge (pap), samp, and beans are popular in several homes. However, the availability of a wide range of nutritious foods may be restricted due to economic limitations. According to Oldewage-Theron and Kruger [8], low-income households frequently depend on inexpensive, calorie-dense meals that include high levels of refined carbohydrates and harmful fats but are deficient in key nutrients. The dietary pattern is influenced by cultural preferences and financial variables, with the socioeconomic condition of households directly impacting food choices. In addition, Battersby [9] emphasises that food insecurity in South Africa is primarily caused by economic inequities, resulting in limited availability of affordable fresh fruits, vegetables, and meats for poorer families. When creating interventions to improve the school food environment (SFE) and address malnutrition in South African schools, it is important to carefully evaluate the intersection of cultural traditions, economic availability, and food accessibility.

The school food environment (SFE) refers to the physical, social, economic, and policy-related factors within and around schools that influence learners’ food choices, dietary behaviours, and overall nutrition [10]. SFEs are multi-faceted and affected by a variety of factors, including physical facilities, SFE stakeholders (learners, teachers, school management, parents, food service personnel, external vendors), and the wider society at large [11].

In South African public schools, food availability and accessibility are facilitated through the National School Nutrition Programme (NSNP), tuckshops, learners’ lunchboxes, and street vendors [4,12]. The NSNP aims to provide balanced meals that contribute to at least 25–30% of a child’s recommended daily allowance. These meals typically include a combination of staple foods (like maize, rice, or bread), protein sources (such as beans, lentils, or occasionally meat), and vegetables, which follow a one-week cyclic pattern [13]. Globally, there is a strong recommendation for using a comprehensive strategy to promote healthy eating habits at the school level. However, effectively harnessing such a strategy must be informed by or require insights into the challenges and opportunities within the SFE from the stakeholders. In essence, incorporating diverse perspectives ensures that improvements to the SFE are comprehensive, responsive to learner needs, supportive of the overall well-being of learners, and sustainable.

The SFE has a significant impact on learners’ food choices. Studies in high- and middle-income countries show that the SFE influences diet quality and obesity risk [14] and the ability to foster healthy eating behaviours among learners. Given that children spend a significant amount of time in school, where they are likely to consume up to 30% of their daily calories, a healthy SFE is vital [2]. However, studies have shown that the SFE in South African public schools is not conducive to promoting healthy eating and drinking behaviours. Many learners in these schools regularly consume ultra-processed foods high in sugar, salt, and harmful fats [15,16,17,18,19]. The existing SFE seems to encourage dietary patterns that do not adhere to national healthy eating standards, as outlined by the South African Guidelines for Healthy Eating. This might potentially increase the likelihood of children becoming overweight and obese [20,21,22]. Moreover, in under-resourced settings, children often face hunger and food insecurity, leading to undernutrition, which impedes their ability to thrive and thus perpetuates the cycle of poverty and illness [23,24].

Given the significant influence of the school food environment (SFE) on learners’ food choices and the growing prevalence of obesity and undernutrition in South African children, it suggests that enhancing the SFE through specific interventions can positively influence learners’ food choices, decrease the consumption of highly processed foods, and alleviate the triple burden of malnutrition in South African public schools. The significance of this research lies in its ability to guide policy formulation by providing evidence-based recommendations for improving the SFE. This, in turn, could promote healthier dietary practices, lower the likelihood of non-communicable diseases, improve cognitive abilities, and enhance academic performance. Ultimately, these outcomes could lead to improved public health results and help break the cycle of poverty and illness.

Therefore, this study aimed to gain a deeper understanding of learners’ perspectives as key stakeholders in the SFE, explore the link between the SFE and their food choices, and identify the barriers and enablers to fostering a healthy SFE in South African public schools. This formative enquiry of the SFE stakeholder perspective formed part of “Modelling the Blueprint to Improve the School Food Environment in South Africa”, a project supported by the UNICEF, the South African Department of Basic Education (DBE), and the Department of Health (DOH), which aimed to improve the SFE in South Africa by implementing evidence-based interventions to reduce the triple burden (overnutrition, undernutrition, and micronutrient deficiencies) of malnutrition. We hypothesise that improving the SFE through learner-driven insights will significantly influence their food choices by reducing the consumption of unhealthy foods and increasing access to healthier options.

## 2. Materials and Methods

### 2.1. Selection of Schools

This study was conducted in the KwaZulu-Natal (KZN) province, South Africa, in the iLembe district. In this district, a total of six public schools—three primary (Grade R to Grade 7) and three secondary (Grade 8 to Grade 12) schools—representing an urban and rural mix and national quintiles (NQ) 1–5 schools were preselected by the KZN Department of Education to serve as pilot schools. In South Africa, NQs are used to stratify public schools. Quintiles 1 and 5 corresponded to poorer and higher socioeconomic categories, respectively. Schools within quintiles 1 to 3 provide schooling to underprivileged learners and are exempt from school fees [25].

### 2.2. Study Design and Recruitment of Participants

This study utilised a qualitative methodology to explore learners’ viewpoints regarding their food choices within the SFE. This approach was appropriate to leverage the findings to inform the design of interventions, as qualitative methodologies are beneficial in offering valuable perspectives and information in this regard [26]. Semi-structured focus groups were used for the interviews because they provide a naturalistic setting where participants can be open and honest.

Participants were selected from learners in Grades 6 to 7 in primary schools and Grades 10 to 12 in high schools specifically for enhanced comprehension of questions. At the start of this study, the schools were sensitised, and this study was explained to the principals. The selected schools circulated participant information sheets, which were provided by the researchers and included parental consent forms. Additionally, informed consent and assent were collected from learners. Every focus group was intentionally structured to have a diverse representation of genders. A mixed group was selected to better represent the opinions of the larger group of learners.

The FGD interview guide for this study was developed by the researchers through four essential steps: identifying research objectives, selecting domains with specific questions, developing the guide, and pilot testing to ensure that the data collected were rich, relevant, and aligned with the research objectives. In the first step, this study’s objectives (understanding the learner’s perspective as a key stakeholder in the SFE and examining the link between the SFE and the learner’s food choices) were clearly defined. In the second step, the researchers developed domains (broad categories) with specific questions aligned with the study objectives using relevant literature. In step three, the FGD guide was developed using open-ended questions for each domain to encourage detailed responses and allow participants to freely share their experiences, opinions, and perspectives. Probing questions were included to explore interesting or unexpected topics in greater depth, ensuring that the guide was flexible enough to adapt to the flow of the conversation. The questions were organised in a logical sequence, progressing from broad topics to more specific concepts. The domains of the questions included the perception of healthy foods, practice of nutrition and feeding, health issues, food availability in and around school, the practice of breakfast consumption, formal (tuckshop) and informal vendors (a vendor who sells outside the school boundary), marketing of unhealthy foods, challenges in accessing healthy foods, physical activity, nutrition education, and WASH (Water, sanitation and hygiene) practices in school, and so on (See Supplementary File S1). The questions included in this study were designed to be open-ended to minimise the use of leading questions and to prevent learners from providing simple yes or no answers. The researchers played the role of a moderator, aiming to guide the discussions in a manner that allowed the learners to take the lead. The measures used included the provision of assurances to learners on the anonymisation of direct quotes, the clarification that there were no definitive correct or incorrect responses, and the use of indirect questions. In step four, the FGD guide was pilot-tested with a small group of learners similar to the study sample to identify any issues with the questions or flow and to refine the guide accordingly. Feedback from the pilot test was used to revise the guide.

### 2.3. Focus Group Discussion (FGD) Procedure

Focus group discussions (FGDs) were performed in person with a group of four to eight learners. The FGDs were conducted between March 2023 and April 2023 in a quiet room during the school day. An overview of the research was given at the beginning of each FGD. The group size of 4 to 8 learners for each FGD was purposefully chosen to ensure meaningful participation from each learner and to foster an environment conducive to open, comfortable dialogue. Smaller group sizes are often recommended in qualitative research with children to allow all participants to contribute and ensure that quieter or more hesitant learners are given the space to express their views and prioritise the depth of insight over the number of participants. Smaller groups allow for more in-depth discussions and better management of group dynamics, especially when discussing potentially sensitive topics such as food insecurity. The focus was on creating a child-friendly atmosphere where learners felt comfortable sharing their experiences and perspectives openly.

To establish rapport and relieve fear, techniques such as using age-appropriate language and participating in icebreaker activities were used. Sensitive themes, such as food insecurity, were introduced gradually, frequently utilising narrative or hypothetical scenarios to allow children to voice their opinions indirectly. The emphasis was on active listening and promoting participation from all children, making them feel heard and appreciated. The relationships between researchers and children were handled by establishing trust and reducing the perception of authority, allowing the children to feel more at ease. Ethical considerations, including informed permission and confidentiality, were strictly followed, creating a safe environment for learners to express their experiences and ideas. The number of FGDs conducted was predetermined by qualitative research best practices and guided by the principle of saturation. A systematic review by Hennink and Kaiser suggests that data saturation is typically reached after 4 to 8 focus group discussions [27]. Six FGDs were conducted for six schools, each lasting approximately one hour after finding that discussions were similar and reached data saturation. In our study, after conducting six FGDs, we found that the data had reached saturation, meaning additional focus groups would not have provided significantly new insights.

The FGDs were all audio recorded, verbatim transcribed, and anonymised before analysis. During the anonymisation process, unique identifiers (such as learner one and learner two) were substituted for the learners’ names, and care was taken to remove any information that could be used to identify other people or locations.

### 2.4. Data Analysis

The data were evaluated with an inductive theme approach, as outlined by Braun and Clarke [28]. Researchers independently coded the same transcripts, compared their codes, and discussed any discrepancies at regular meetings. Through these discussions, a consensus on the most appropriate codes and themes was reached. To maintain quality, the transcripts were carefully examined multiple times, and similar representative quotes were categorised and assigned codes. During each round of analysis, codes were established, modified, and organised into themes. Finally, themes were evaluated, recreated, and refined collectively for all schools until only a few distinct, data-rich themes remained.

## 3. Results

Six FGDs were conducted with learners from each of the participating schools. The demographic profile of learners who participated in the FGD is represented in Table 1.

The thematic analysis process identified fourteen themes, and several subthemes emerged from the learner FGD, as reflected in Table 2 with their representative codes. NQ denotes the school quintile ranging from 1 to 5. If no NQ is specified, the representative quotes pertain to all schools. Subthemes were further categorised as a barrier (−), enabler (+), or both barrier and enabler of the SFE (±).

### 3.1. Individual Level Considerations to Food Choice

#### 3.1.1. Autonomy in Food Choice

Learners highlighted personal preference as a significant factor in determining their food choices. While learners reported consuming traditional and healthy food such as boiled chicken, bread, rice, and vegetables like spinach, peas, green pepper, and carrots, their preferences leant towards unhealthy foods, including fried chips, yababa (Russian sausage), vetkoek (fried dough bread), ice blocks, and fizzy drinks. There was a noticeable disparity in the consumption of unhealthy food across schools between the lower and higher quintiles. In schools classified as NQ1, inexpensive sweets and flavoured water ice were the most popular choices, but in NQ4 and NQ5 schools, costly foods such as yababa and vetkoek were preferred. Learners reported that they also preferred to consume fruits like oranges, apples, pineapples, strawberries, salads, water, and roasted chicken. Interestingly, the investigation also revealed that learners attending rural schools preferred traditional meals such as phutu, meat, and samp. In contrast, learners from peri-urban or urban settings were more inclined towards choosing highly processed foods like pies, pizza, hot dogs, and cakes.

#### 3.1.2. Social Perception of Healthy Foods

Most learners demonstrated a good understanding of healthy foods. Learners recognised the benefits of consuming water, fruits, and vegetables and believed that a balanced diet should include carbohydrates, proteins, vitamins, and minerals. However, they pointed out that certain fruits are quite costly, and the availability, accessibility, and variety were limited within the SFE.

#### 3.1.3. Hyperpalatable Foods and Beverages

The learners claimed that diets containing excessive amounts of salt, sugar, and oil, such as fried chips and vetkoek, are harmful to health. Despite this, one learner pointed out that their generation considers fruits and vegetables less desirable or unhealthy when compared to hyperpalatable foods.

### 3.2. Food System Influence on Eating Habits and Practices

#### 3.2.1. Adaptive Food Preferences

Generally, learners reported a preference for traditional home-cooked meals such as samp and beans, rice, mashed potatoes, maize meal, meat and beans, Biryani, canned fish curry, canned beans, phuthu, and green leafy vegetables cooked with ground peanuts. However, learners in higher quintile schools tended to prefer highly appetising processed and convenience foods like pies, pizza, chips, fried chicken, and vetkoek.

#### 3.2.2. Palatability of Fruits and Vegetables

Learners demonstrated a good understanding that fruits and vegetables are beneficial for their well-being. They have a preference for fruits, including pineapple, strawberries, oranges, and others, possibly due to their appealing flavour. Many learners reported that vegetables are not palatable due to their taste and texture. Yet, some learners enjoyed certain vegetables, such as sweet potatoes and carrots.

#### 3.2.3. Multiple Food Access Points Driven by Taste and Need

Learners have different food access points in the SFE. They consume meals both from the NSNP and the school tuckshop. Other learners consumed the NSNP meal only when their desired meal was prepared. Additionally, participants in the FGDs revealed that some learners purchase food from informal vendors. A small minority of food-insecure learners abstained from the SFP due to concerns about potential food allergies, and some learners chose to bring packed lunches from their homes.

#### 3.2.4. Impact of Food Labelling on Food Choices

A few learners indicated that they use the food label to read the date of manufacture and expiration, but most learners stated that they needed help reading the entire label. Yet, some learners who are extremely conscious of their health use the food label to evaluate the amount of calories included in food products. Some learners additionally verify the presence of preservatives in a food label. Some learners only examine food labels when they have reason to doubt the quality of a specific food item and are curious about its ingredients.

#### 3.2.5. Sensory Perception of Food

Learners are able to differentiate between foods that are high in sugar and fat through the use of their sensory perception.

#### 3.2.6. Ultra-Processed Food Consumption

Although most learners acknowledge consuming processed food like polony and cheese, the FGDs demonstrate their lack of understanding of what ultra-processed foods are made of. However, several learners expressed concerns over the negative effects of excessive intake of ultra-processed food on their health.

#### 3.2.7. Water Provisioning and Consumption

Most learners stated that the drinking water provisioning at their school was safe. Our research revealed that the learners demonstrated self-motivation and adhered to the recommended daily water consumption. Additionally, a number of educators actively promoted drinking an adequate amount of water daily.

#### 3.2.8. Unequal Food Accessibility

The main barrier to accessing food is an individual’s financial situation. Most learners conveyed their inability to purchase their preferred food due to elevated prices; however, some learners accessed their preferred food due to their higher purchasing power. Regardless of NQ, all learners experienced this situation.

### 3.3. Consciousness of Health Issues and Outcomes

#### 3.3.1. Adoption of a Healthy Lifestyle

Most of the learners showed a clear understanding of a healthy lifestyle and personal well-being. Keeping healthy and avoiding illness were considered by some learners to be the two most crucial aspects of personal well-being. It is clear from their responses that they were aware of the health advantages of eating vegetables and the negative consequences of consuming unhealthy foods.

#### 3.3.2. Awareness of Unhealthy Diet and Health Outcomes

The learners were aware of the adverse impact of consuming food that contains excessive amounts of sugar and salt. Certain learners highlighted the connection between poor lifestyle choices and obesity, food allergies, heart diseases, and diabetes. It was noticeable that when questioned about “healthy eating”, most learners only mentioned healthy dietary choices, such as vegetables. They believe that a diet that is exceptionally high in ultra-processed foods might result in obesity and issues associated with cardiovascular disease.

### 3.4. Food Provisioning and Foods for Sale in the SFE

#### 3.4.1. Institutionalised Provisioning of Food—NSNP

The NSNP receives mixed feedback from learners across different school quintiles. Learners from lower quintile schools appreciate the programme, noting its importance for those from poor households. However, some learners expressed dissatisfaction with small portion sizes. In higher quintile schools, participation varied based on the meals served, with some learners choosing to eat only when certain foods were served. There is a general appreciation for the programme, though suggestions for improvements are mentioned. Overall, the programme was seen as beneficial; however, room for enhancing meal variety and portion sizes was highlighted.

#### 3.4.2. Personal Provisioning of Food—Lunch Boxes

The practice of bringing lunches to school was limited to a few learners. Our observation revealed that most of the packed lunches brought by the learners consisted of unhealthy foods such as white bread with fillings like cheese, bacon, or polony. Schools did not have any guidelines regarding the contents of packed lunches.

#### 3.4.3. Personal Provisioning—Tuckshops

School tuckshops offer a variety of foods, predominantly unhealthy options, such as packaged chips, vetkoeks, candies, fizzy drinks, and fried food items. Unhealthy foods are often purchased because they are more desirable to learners and less expensive than healthier ones. The learners unanimously expressed dissatisfaction with the limited variety of food options provided at the school tuckshops, highlighting the absence of healthy food choices. However, there was a clear indication that schools in the higher quintile offer relatively costly unhealthy food options such as biryani and hot dogs. In contrast, lower quintile schools have a higher preference for items like biscuits, sweets, and chips. This demonstrates the disparity in purchasing power among learners. Some learners desired the school tuckshops to provide a broader range of fruits and nutritious choices, such as sandwiches.

### 3.5. Practice of Breakfast Consumption and Participation in Institutionalised Breakfast Programme

Learners’ experiences with breakfast varied widely across different school quintiles. Some learners consumed breakfast regularly at home, eating items like porridge, cornflakes, and Weetbix. However, many learners reported often going to school on an empty stomach due to a lack of food at home. In schools that have a breakfast programme, the availability of breakfast at school, such as breakfast porridge, was appreciated and seen as beneficial, especially since many learners arrived at school hungry. In schools that did not have a breakfast programme, learners expressed willingness to participate in a breakfast programme, highlighting the positive impact it could have on their concentration in class.

### 3.6. Other Entry Points to Food Access

#### Determinants of Food Purchasing from Vendors

Learners who participated in the FGDs indicated that they regularly buy food from formal and informal vendors. When purchasing food from vendors, learners consider aspects such as cost, cleanliness, food safety, and the vendor’s reputation. However, some learners overlook other considerations and prioritise satisfying their hunger over the source of the food. Common purchases included foods like vetkoek, fried chips, sweets, hot dogs, and packaged crisps, with prices ranging from R1 to R15.

### 3.7. Exposure to Marketing Around School

Across all schools, a number of learners reported finding food marketing posters inside their school premises and across two primary schools, learners reported participating in a food sample testing event organised by a food company.

### 3.8. Barriers and Facilitators of Accessing Healthy Foods

#### 3.8.1. Socioeconomic Factors

Learners felt that their purchasing power as school-aged children was limited, and most learners come from disadvantaged communities, enabling them to purchase cheaper food, such as sweets, as healthy foods are too costly for them to afford.

#### 3.8.2. Environmental Exposure

According to the learners, healthy foods such as fruits and vegetables are less palatable and difficult to store for extended periods. In contrast, learners who owned a vegetable garden had access to a wide variety of fruits and vegetables. Learners identified taste, satiety, and cost as their primary constraints and obstacles to eating healthy foods. However, learners might be willing to consume healthier food choices, such as fruits, if they were more cost-effective and readily accessible in their SFE.

### 3.9. Participation in Physical Activities

#### Barriers to Promoting Physical Activity

School curricula include physical education. Some learners stated that they exercise by engaging in activities such as football, netball, and indigenous games. Despite this, most learners expressed dissatisfaction with the existing infrastructure and a need for more equipment to engage in physical exercise, particularly after COVID-19. However, learners expressed an interest in attending a sports day at their respective schools.

### 3.10. Exposure to Food and Nutrition Literacy

#### Sources of Nutrition Education

Both primary and secondary schools offer subjects that include a curriculum on food and nutrition. As a result, learners are familiar with some fundamental information on nutrition, wellness, and diseases. Specifically, learners highlighted the significance of the “Life Orientation” subject in enhancing their understanding of nutrition. Furthermore, learners reported access to other sources of nutrition knowledge outside their school curriculum, such as books and the internet. Occasionally, family members and local clinics also provided them with information on healthy cooking and living.

### 3.11. Institutional Support for a Healthy Lifestyle

#### Availability of Health and Nutrition Activities and Services

Some schools have shown support for promoting a healthy lifestyle. Learners also expressed their teacher’s assistance and encouragement in adopting a healthier diet. However, it was apparent that the food environment did not facilitate the adoption of healthy eating and drinking practices. Some schools lacked municipal support for providing clean water. In addition, schools often did not have other health-related services and infrastructure, although there is supposedly one school with a Care and Support office. Meanwhile, some schools prioritised the promotion of mental well-being. However, schools often lacked the infrastructure to facilitate health and nutrition initiatives. Learners expressed interest in participating in physical activities and making healthy food choices, provided schools had more physical education equipment, and tuckshops offered nutritious food and beverage options.

### 3.12. External Influence on Health Information

#### Influence Outside the School

Learners were influenced by internet platforms, books, family members, and healthcare practitioners while seeking health-related knowledge. Specifically, the impact of parents on making healthy eating choices had an important impact. Learners constantly encountered advertisements for food and drinks via various media channels such as television, posters, and social media. These advertisements are very appealing to the learners and have a significant impact on their food and beverage choices.

### 3.13. Water, Sanitation and Hygiene (WASH)

#### Safe or Unsafe

Some learners complained that the water supply in schools was unsafe and insufficient regarding cleanliness and sanitation. Some learners expressed dissatisfaction with the need for more cleanliness in water tanks and inadequate toilet facilities. Furthermore, it was evident that NQ1 and NQ2 schools primarily relied on rainwater harvesting as their primary source of drinking water. On the contrary, some schools demonstrated impressive waste management practices. In addition, learners possess a strong understanding of personal cleanliness and behaviours, and they believe that these practices will be encouraged via school activities.

### 3.14. Internal School Physical Environment

#### Maintenance and Upkeep of the Physical Environment

While the learners found the environment satisfactory, they expressed concern about the physical threats in their surroundings. For example, old and rusty playground equipment.

## 4. Discussion

**Figure 1:**
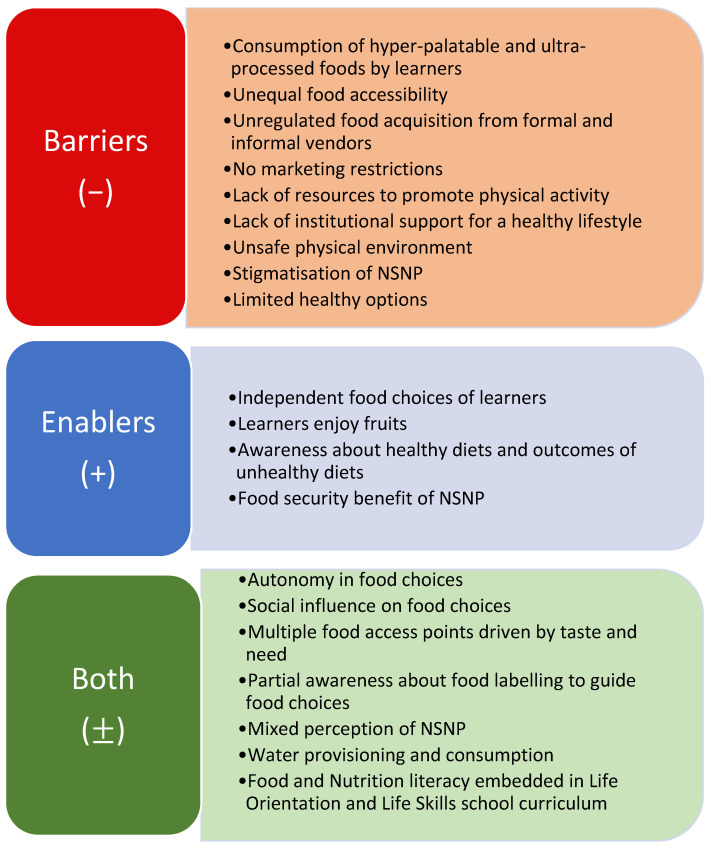
Barriers and enablers to a healthy SFE.

This study clearly demonstrates that certain factors serve as barriers to making healthy eating choices in the SFE. In addition to potential barriers, there are factors that may either promote or hinder the development of a healthy SFE (Figure 1). Challenges to making healthy food choices among learners include consuming very appealing and heavily processed foods, unequal access to food, and unregulated food purchases from both formal and informal vendors. Furthermore, the lack of restrictions on marketing, inadequate resources for promoting physical exercise, and insufficient institutional support for a healthy lifestyle intensify the problem. The presence of an unsafe environment and the stigmatisation of the NSNP impede the adoption of healthy eating habits, in addition to the lack of healthy food choices. However, there are factors that can facilitate the adoption of healthier food options, including learners’ autonomy in selecting their own food, their liking for fruits, and their understanding of the advantages of a healthy diet and the consequences of poor eating behaviours. The NSNP also offers food security advantages. Factors such as autonomy in food choices, social influences, and access to multiple food outlets driven by taste and need can either inhibit or facilitate food consumption. Although there is some knowledge of food labelling that may provide guidance for making choices, opinions about the NSNP are diverse. Water supply and usage, as well as the integration of food and nutrition education into the Life Orientation and Life Skills curriculum, are essential in influencing healthier food environments.

### 4.1. Barriers to a Healthy School Food Environment

Food accessibility and availability in South African public schools are provided by the NSNP, tuckshops (formal vendors), learner lunchboxes, and street (informal) vendors [4,12].

Our research demonstrates that learners possess autonomy in their food choices, which may serve as indicators of an individual’s personal identity, group association, and cultural identity. Both family and social networks influence food choices. This is because individuals observe and take into account what others choose to eat. They also engage in negotiations with others when it comes to shared food and get support or lack of support from others in making their preferred food choices. Adolescents, for instance, may eat “junk food” as a means of indicating their connection with their peer group, while healthy food serves as a symbol of family [29]. Previous studies revealed that many learners were aware that low-sugar and low-salt foods are healthier. Yet, their selections of school food items often had excessive sugar or salt content. School food vendors offer less healthy food options, which may limit learners’ eating choices [30]. Another research by Magalhaes et al. [31] found that learners eat HFSS (high fat, salt, sodium) food due to their inability to control their food cravings when they smell and see tasty food and have a lack of self-motivation to eat healthier. It is also considered that cultural preferences influence food intake; however, young people favour convenience and taste over health advantages [32]. Children are often unaware that their diet impacts their development, feelings, and behaviours [33].

Food from the school tuckshop and formal and informal vendors inside and outside the school were other key access channels for unhealthy foods. Learners purchased food from vendors every day based on pricing and selection/variety. Some individuals prioritised hygiene when selecting a food vendor, while others demonstrated indifference to other considerations. HFSS food and a few fruits were offered at varied prices. This research confirms previous findings that identify the relative convenience of access and availability of unhealthy diets as a barrier for young learners. This ease is described by factors such as time, cost, facilities, and the presence of unhealthy food. According to a qualitative research study in South Africa, participants said learners often purchased food items from vendors due to the unappealing taste of the food supplied at schools [34]. Okeyo et al. [30] found that learners either consumed school-provided meals via the SFP, purchased meals from the tuckshop or informal vendors, or brought lunches from home. Having food from both the vendors and the school might cause overeating, which can ultimately result in obesity and overweightness. A study examining the relationship between BMI and food intake found that learners who carried their lunch boxes to school had a lower BMI than those who bought food from school tuckshops [35]. Hence, improving the nutritional quality of the food available in the school tuckshops will substantially improve the learners’ overall dietary habits.

The media also greatly influences children’s perceptions of nutrition [36]. Various food sector marketing strategies are used in schools, and some tuckshops use basic promotional methods to promote their products. Studies revealed that children’s tastes, purchasing patterns, and consumption patterns for various categories of food and drink and product brands are influenced by food advertising. Advertising in schools involves various forms, such as posters, signage, logos, and brand names displayed on food and beverage containers, vending machines, and accessories. It also includes promoting food sales as fundraisers, corporate sponsorship of events, advertising in school publications, and providing corporate-sponsored classroom curricula and scholarships [37]. Recent research also suggests that social media has a greater impact on teenage motivations and behaviours than conventional parenting methods [38]. Erzse et al. [39] found that South African learners face challenges from aggressive marketing of unhealthy foods and drinks. Several decision-makers cited a trade-off between profit and product quality to explain the lack of healthy alternatives in tuckshops.

A lack of health infrastructure and inadequate support for physical activities are other obstacles to achieving a healthy lifestyle. Rural schools are remote and undeveloped. Some schools lack basic physical infrastructure and resources [40,41]. However, South African schools provide health care. School Health Programme implementers highlighted a shortage of school health teams as a reason for failing to meet objectives [42].

Learners were generally aware of personal hygiene practices and waste management techniques. Our investigation shows that some schools have physical dangers like rusty playing equipment, etc. Learners are usually happy with the school’s physical surroundings, which significantly affects children’s hygiene practices [43].

Understanding learners’ perspectives on their food choices and the SFE is crucial for developing effective interventions and policies to improve the SFE. By addressing the barriers identified in this research—such as the availability of unhealthy food options, marketing and sponsorship by the food industry, the influence of social networks and media, and the lack of adequate health promotion infrastructure—schools can create a healthier and more supportive environment for learners. This will enhance their physical well-being and positively impact their academic performance, behaviour, overall development, and quality of life.

### 4.2. Enablers of a Healthy School Food Environment

With the many obstacles that impede the establishment of a healthy SFE, there are a number of enablers that, when used strategically, might help in producing the intended outcomes and fostering a healthy SFE. An individual’s food system plays a crucial part in comprehending the many elements that impact their food preferences, as well as the effect of culture in shaping those preferences. Food behaviours are mostly acquired via the transfer of knowledge from parents to children, which greatly influences the acceptance of food [29]. According to a research study, most teenagers eat healthily at home. However, on school days, learners often neglect to bring their lunch boxes with them to school [36].

For the most part, learners ate from the NSNP, often called the school feeding programme (SFP) worldwide. The NSNP serves a learner population of over 9.6 million learners, which includes secondary schools. The NSNP is a primary provider of nutrition for many learners, particularly those from socioeconomically disadvantaged households [44]. According to the DBE (Department of Basic Education, South Africa), the NSNP is a significant government policy aimed at reducing poverty. School meals serve as an essential support network for at-risk children and their families [45]. According to Mafugu [46], the NSNP helps with varying dietary preferences across schools. Our study found that the NSNP alleviates the burden on low-income families by providing breakfast (limited schools) and lunch for learners. Due to financial constraints and limited time, a majority of the learners skipped breakfast before school. Also, the majority of learners do not see culture or beliefs as a barrier to consuming NSNP. Hochfeld et al. [47] recommend offering breakfast daily before school starts to enhance attentiveness and classroom engagement. Our research indicates that learners would appreciate an SFP that includes breakfast for those who arrive hungry. However, this study found that learners had autonomy in choosing their food and acquired food from multiple school food access points in the SFE. The SFP were the primary source of lunch for learners. Though some learners did bring their packed lunches to school, the majority did not, and schools should have established policies to address this. According to a study, a significant proportion of learners in Cape Town, South Africa, failed to bring nutritious items from their homes. The reasons for the lack of nutritional information or particular preferences among mothers and learners are unknown. Nevertheless, it was apparent that, overall, learners had knowledge about which foods were more nutritious; however, this knowledge did not influence their purchasing action [48]. Our research also confirms that learners possess the ability to distinguish between healthy and unhealthy foods and are aware of the adverse health consequences associated with a poor diet. However, no correlation was found between nutritional knowledge and the adoption of healthy eating habits. Learners prioritised the taste of fruits above health advantages, whereas vegetables were the least chosen. Brown et al. [49] discovered that teenagers were ignorant of the recommended daily dietary fibre intake and fruit and vegetable serving sizes. The inability of learners to read food labels is a significant problem since understanding food labelling influences dietary choices. Very few learners read food labels due to difficulty comprehending nutritional information. Xazela and Chinyamurindi [50] propose that not reading nutrition labels may be due to misunderstanding, misinterpretation, or lack of initiative in understanding the information. Our research revealed that certain learners were interested in reading food labels, particularly for shelf-life, level of preservatives, and macronutrient composition.

The initiation of health education and the promotion of a healthy lifestyle need to start during childhood and consistently continue into kindergarten and schools with the provision of evidence-based interventions [51]. In South Africa, school syllabuses in both primary and secondary public schools cover nutrition in particular subjects. While the curriculum includes some nutrition education, more is needed to promote a change in behaviour among the learners [52]. Access to food and health information is enhanced by widespread internet access, internet search engines, and social media [53]. Therefore, fostering nutrition knowledge in children may lead to healthier eating habits and improved health and nutrition status.

Learners stated that having vegetable gardens in their homes may promote healthy eating habits. According to research by Payán et al. [54], teenagers obtained fruits and vegetables from local markets, including their homes and schools. However, they found that healthy foods like fruits and vegetables were more expensive than the unhealthy ones in their neighbourhood. The study also showed that most learners were self-motivated to drink the daily required water, whereas weather influenced some. Schools have drinking water, but safety and quality were problems. The Development Bank of Southern Africa [55] states that inadequate clean water negatively impacts learners’ academic performance and attendance. Lack of safe drinking water may lead to illness, dehydration, and poor hygiene standards for learners. School programmes cannot be run without water and adequate sanitation facilities for learners and teachers. This harms the school system by limiting growth and promotion opportunities.

Learners emphasised good nutrition and exercise for a healthy lifestyle. They noted obesity and respiratory illnesses among family and friends. Lifestyle choices might cause health issues. Increased awareness of illness signs leads to increased preventative actions and health checks. A lack of understanding about diseases and screening/treatment choices constitutes a considerable health risk [56].

Recognising and leveraging the facilitators within the SFE is essential for promoting healthier eating habits among learners. These facilitators, including the influence of family and social networks, the provision of nutritious meals through the NSNP, and the integration of health education in school curricula can significantly contribute towards the creation of a healthier SFE. By addressing the existing barriers and strategically utilising these facilitators, schools can foster a culture of wellness, improve dietary habits, and support learners’ overall development and well-being. Collaborative efforts among educators, parents, policymakers, and food service providers are crucial in implementing effective interventions and ensuring all learners can access nutritious, safe, affordable, and sustainable food and beverage options.

Ensuring a healthy SFE is a long-term investment that empowers learners to make informed decisions about their health. It sets the foundation for lifelong well-being, making the SFE a cornerstone for promoting lifelong health and well-being.

### 4.3. Strengths and Limitations of This Study

This study’s merits lie in its comprehensive analysis of food service offers, its incorporation of schools serving learners from five distinct socioeconomic statuses, its use of national standards to define wholesome food, and its investigation of the accessibility to healthy and unhealthy food options. This setting is a potential environment for the delivery of nutritional interventions, as schools provide learners with continuous contact time. School-based interventions are economically viable and provide an opportunity to reach a wide range of learners, regardless of their socioeconomic background or ethnicity. The relatively limited sample size may be regarded as a limitation. However, the schools selected from each NQ are representative of the public schools in South Africa.

## 5. Conclusions

The findings of this study highlight the significant barriers and enablers within the SFE in South African public schools, as perceived by learners. Key barriers include the prevalence of unhealthy food options from both formal and informal vendors, the aggressive marketing of unhealthy products, and insufficient regulation of these practices. Learners also identified the stigma surrounding the NSNP and the lack of variety in its offerings as factors limiting its effectiveness. However, this study also uncovered positive aspects of the SFE, including learners’ autonomy in making food choices, their preference for fruits, and their awareness of the benefits of a healthy diet. The NSNP plays a crucial role in providing food security, particularly for learners from socioeconomically disadvantaged backgrounds. Promoting healthier food choices can be facilitated by improving nutrition education, enhancing the NSNP, and regulating food marketing within the school environment. By addressing these barriers and leveraging identified enablers, schools can create an environment that supports healthier food choices, which could positively impact learners’ well-being, academic performance, and development. This study underscores the importance of learner involvement in shaping interventions aimed at improving the SFE. Empowering learners through nutrition education and policy changes can foster more informed food choices, ultimately contributing to a healthier SFE.

## Figures and Tables

**Table 1:** Demographic profile of the learners.

School	Age Range (in Years)	Gender (*n*)		Gender (%)	
		Male	Female	Male	Female
NQ1 Primary School (Grade 7)	13–14	3	3	50	50
NQ3 Primary School (Grade 6)	12–13	3	3	50	50
NQ5 Primary School (Grade 7)	13–14	0	8	0	100
NQ2 Secondary School (Grade 11)	16–17	3	3	50	50
NQ3 Secondary School (Grade 12)	17–18	3	3	50	50
NQ4 Secondary School (Grade 10)	15–16	1	3	25	75

**Table 2:** Thematic Analysis and Key Quotes from Learners on the School Food Environment.

Theme	Subtheme	Example of Quotes
**Individual-level** **considerations to** **food choice**	Autonomy in food choice (±)	*“We consume too much junk food, for example, vetkoek (deep fried dough bread) (NQ3), pies (NQ4), Yababa (Russian sausage) (NQ4), burgers (NQ4), sweets (NQ1 and NQ3), fried chips (NQ2, NQ3 and NQ4), pizza (NQ5), hot dogs (NQ4), ice lollies (flavoured and sweetened water ice) (NQ1), cakes (NQ5), fizzy drinks (NQ2), and energy drinks (NQ2)”* *“We enjoy fish and meat (NQ1), boiled chicken”* *“I like vegetables like mixed vegetables, peas, carrots, cabbage, spinach and green pepper” (NQ5) * *“A simple roasted chicken without spices and fruits such as oranges, apples, pineapples, and strawberries” (NQ5)*
	Social perception of healthy foods (+)	*“Fruits are healthy, but they are not easily accessible because we do not have a lot of people selling them in our area. Even the one who is selling them sells only apples, there just is no variety, and they are expensive” (NQ3)* *“Green leafy vegetables are healthy and they are easily available as most households in this community have their own vegetable gardens” (NQ1 and NQ2)* *“Home-cooked meals are healthy” (NQ3)* *“Drinking water is healthy” (NQ2)* *“In order to eat healthy food, most of us need to balance our diets by including carbohydrates, proteins, as well as vitamins and minerals” (NQ5)*
	Hyperpalatable foods (−)	*“Food with too much oil, salt, or sugar are unhealthy” (NQ1, NQ2 and NQ3)* *“Fruits and vegetables are viewed as unhealthy foods by people in our generation” (NQ5)*
**Food system** **influence on** **eating habits and** **practices**	Adaptive food preference (±)	*“Rice (NQ2, NQ3), chicken and salad (NQ3) meat (NQ1, NQ2) and beans (NQ2), Briyani (seasoned rice with meat/vegetables) (NQ2), fried meat (NQ2), samp (dried corn kernels cooked into a stew)(NQ2), canned fish curry (NQ2), canned beans (NQ2), phuthu (steamed crumbly maize meal) with green leafy vegetables cooked with ground peanut (NQ1), boiled chicken with Dombolo (steamed bread) (NQ1), chips and bread with cheese (NQ3), fried green beans (NQ5)”* *“I like roasted chicken and pizza” (NQ5)* *“Yoghurt or fruit salad” (NQ4)* *“My favourite is cheese sandwich with lettuce and cucumber” (NQ3)* *“I enjoy chicken and pizza” (NQ5)*
	Palatability of fruits and vegetables (+)	*“I like fruits and vegetables because they are healthy”* *“I only like fruits not vegetables, I just do not like how vegetables taste” (NQ2, NQ5)* *“Fresh sweet potatoes from the garden are what I prefer” (NQ5)* *“I do like certain fruits” (NQ3)*
	Multiple food access points driven by taste and need (±)	*“I eat from both the NSNP and the school tuckshop” (NQ2)* *“Twice a week I benefit from the NSNP, those are the days when phuthu and beans or rice and fish is cooked” (NQ1)* *“I buy fried chips and vetkoeks from the formal vendors and then buy scones from the informal vendor” (NQ2)* *“I bring my own lunch” (NQ3)* *“I did not eat from the school kitchen because of my food allergies” (NQ5)* *“I buy pies from the formal vendors almost every day” (NQ3)*
	Impact of food labelling on food choices (±)	*“I do not check the labels, as long as the packaging is attractive, I buy the food” (NQ1)* *“We do not read food labels because we do not understand much about them” (NQ2)* *“I check the expiry date and the amount of energy it contains” (NQ3, NQ5)* *“I read whether or not they include preservatives” (NQ5)* *“Most of the time, I don’t read the labels, but when I don’t trust what’s inside of the product, I do” (NQ5)*
	Sensory perception about food (±)	*“Food with too much oil is easily visible because even after eating you can see traces of oil on the container, but on the salt and sugar part; you would have to taste to know if food does contain high amount of sugar or salt” (NQ1, NQ2, NQ3), * *“Sugary food is sticky” (NQ2, NQ3)*
	Ultra-processed food consumption (−)	*“Yes, I do consume a variety processed foods” (NQ1, NQ2, NQ3)* *“I do not know what the content of this ultra-processed foods is” (NQ2, NQ4)* *“There are some that I do eat, but I do not eat much because I know I might get sick” (NQ3)*
	Water provisioning and consumption (±)	*“Most of the times, we do have safe drinking water” * *“There is nothing encouraging us to drink water, I am just self-motivated, and I drink close to 2 litres of water” (NQ2)* *“Only our Natural Science teacher encourages us to drink water” (NQ3)*
	Unequal food accessibility (−)	*“I do not have access to the food I want, issue being my parents cannot always afford the food I want” * *“Yes, I am able to get the kind of food I want, as my mother packs food I requested for my lunch”*
**Consciousness of** **health issues and** **outcomes**	Adoption of a healthy lifestyle. Personal well-being (+)	*“Keeping fit and eating healthy is very important to me”* *“We should balance our consumption of salt and sweets since too much sugar might make you sick”* *“You might get allergies from some foods”* *“I believe that eating more vegetables will help us stay healthy because they contain vitamins and minerals that can help us fight disease”* *“Eating unhealthy food can cause a person to be easily attacked by diseases”*
	Awareness about unhealthy diet and health outcomes (+)	*“If my grandmother eats meat, she gets gout” (NQ5)* *“Eating too much oil blocks your blood vessels and leads to heart failure” (NQ3)* *“Eating too much sugary foods can cause diabetes” (NQ3)* *“Following an unhealthy diet can lead to a person being overweight and being affected by diseases such as heart failure, diabetes, cholesterol or even * *high blood pressure”*
**Food access**	Institutionalised provisioning of food—NSNP (±)	*“I am very happy with this programme as this programme is very helpful to learners who come from poor households” (NQ1)* *“I do not eat food from NSNP” (NQ3)* *“It is dependent on the meal being served per day. I make sure to eat on days when meat is served” (NQ3)* *“I am not happy with the small portion sizes being served” (NQ1)* *We do enjoy the food, but I would prefer if there were more salad options” * *“I think this is a very good programme”*
	Personal provisioning of food—lunch boxes (±)	*“I bring unbuttered white bread with cheese and bacon, cheese only or even polony (Emulsified French sausage) only”* *“No, we all do not bring a packed lunch”* *“No guidelines provided by the school on packed lunches”*
	Personal provisioning—tuckshops (−)	*Packaged crisps (NQ 2, NQ3, NQ5), sugar cane (NQ3), vetkoeks (NQ2), fried chips (NQ2, NQ3), fizzy drinks (NQ2, NQ3), sweets (NQ1), snacks (NQ1), biscuits (NQ1), briyani (NQ4), ice lollies (NQ4), hot dogs (NQ5)”* *“I would be happy if sandwiches were to be sold in this school” (NQ3)* *“They could sell us fruits or vegetable salads in the tuckshop for maybe R 10.00” (NQ5)*
**Practice of breakfast consumption and participation in institutionalised breakfast programme**	Breakfast consumption (±)	*“I have porridge for breakfast” (NQ3)* *“I eat cornflakes, Weetbix” (NQ3)* *“We hardly have any food to consume for breakfast, so I go to school on an empty stomach” (NQ1)* *“If I am not late, yes I do have breakfast in the morning before coming to school” (NQ2)* *“Many learners buy from the tuckshop because they arrive at school hungry” (NQ4)* *“The school provides instant porridge for breakfast and that would mean a lot, as most learners come to school on empty stomachs so it will provide the much-needed meal to start the day” (NQ3, NQ5)* *“I would participate in a breakfast programme if I knew what they are going to prepare for us” (NQ5)* *“I would take part if a breakfast programme is introduced by school since I don’t have breakfast at home and because being hungry makes it difficult to focus in class” (NQ5)*
**Other entry points to food access**	Determinants of food purchasing from the vendors (−)	*“If I do have money; I buy from the vendors” (NQ1)* *“We make use of both the formal and informal vendors”* *“Prices and cleanliness are what I look into most”* *“When I am really hungry, I do not care where I access food, I just want to satisfy my hunger”* *“I buy R2 lollipops and packaged crisps for R2, I buy it almost 4 times a week”* *“Fried chips costs R15”*
**Exposure to** **marketing**	No marketing restrictions (−)	*“There is a poster advertising selling of vetkoeks” (NQ1)* *“In our school, there is a poster there that has noodles, the other day there were people who came here and made a big pot of noodles and served it to everyone” (NQ5)*
**Barriers and** **facilitators of** **accessing healthy** **foods**	Socioeconomic factors (−)	*“It is not easy as healthy food is expensive while unhealthy food is affordable”* *“Banana is expensive compared to sweets, with R1, I can get 2 sweets but with the very same R1, I would be very lucky to even get a banana which is the cheapest fruit”*
	Environmental exposure (±)	*“It is very easy to eat healthy as at home we have avocado trees and fruit trees like banana and oranges” (NQ1)* *“Food like fruits is expensive (NQ2, NQ3) and have a very short shelf life” (NQ3)* *“Since healthy food is not sold in our school tuckshop, we do not have much access to it”*
**Participation in physical activity**	Barriers to promote physical activity (−)	*“We play and gym inside the school premises, we * *do warm-ups, jog and others play soccer and netball and indigenous games. We do this as part of Physical Education Training.”* *“After COVID-19, we no longer have the resources”* *“We get sports day, fun run and excursions where we go out on a trip”*
**Exposure to food and nutrition literacy**	Sources of nutrition education (±)	*“Life Sciences and (Grade 11)”* *“In grade 7 it is taught is Natural Science as well as Life Orientation”* *“Read books at the library”* *“Using the search engines like Google”* *“My mother is the one who actually teachers me how to cook”*
**Institutional support for a healthy lifestyle**	Availability of health and nutrition activities and services (−)	*“The school principal tells us that we must eat healthy food, yet the school tuckshop sells unhealthy food”* *“The school does not put emphasises on Nutrition and what we consume here at school” * *“Yes, because most teachers who come to class always encourage us to drink water and eat healthy”* *“The water in the school is not as clean”* *“We do not have services or infrastructure that supports our health and healthy behaviours” * *“We have the Care and Support office”* *“There are activities that are more focused on mental health”* *“I wish they can be more equipment for physical education which focuses more on our health” * *“I think the tuckshop should focus more on selling healthy food”*
**External influence on health information**	Influence outside the school (−)	*“Internet, Library, Home, Clinic”* *“My mother teaches me about health”* *“I receive health-related information from our local clinic”* *“Parents mainly have an influence on what we eat since they are the ones who have to do the buying, so they choose what we must eat.”* *“Yes, they do, their aim is to get you to buy what they are selling, and they usually win with me because I always want to buy things I have seen advertised”*
**Water, sanitation, and hygiene (WASH)**	Safe or unsafe (±)	*“Our water is harvested from rain” (NQ1)* *“We have rainwater in most water tanks” (NQ2)* *“Sometimes we don’t have water” (NQ5)* *“The water is not safe (NQ1, NQ5) and there are insufficient toilets” (NQ5)* *“Our toilets are not clean” (NQ2)* *“We have dustbins for disposal of waste, we also have a timetable of which class will be on duty to pick up papers at school in the morning and after break and dispose those papers in the right place” (NQ1)* *“Personal hygiene entails washing your body and brushing your teeth”*
**Internal school physical environment**	Maintenance and upkeep of physical environment (−)	*“I am happy as the school is clean” (NQ2, NQ3)* *“The playing equipment is quite old and has rust”* *“The fence has a big hole, and kids get out of the school”*

## Data Availability

Research data are available by writing to the authors.

## Supplementary Materials

The following supporting information can be downloaded at: https://www.mdpi.com/article/10.3390/nu16203542/s1, Supplementary File S1: Focus group discussion guide—learners.
