# Supplementary material for: Stakeholder Perspectives on the School Food Environment: Insights from South African Learners—A Pilot Study
Source: Nutrients. 2024 Oct 18;16(20):3542. doi: 10.3390/nu16203542 (PMC11510002; doi:10.3390/nu16203542)
Supplement: Supplementary file 1 [file nutrients-16-03542-s001.zip › nutrients-3149864-supplementary.pdf]

Supplementary File S1
Focus group discussion guide- Learners

Welcome, and thank you for volunteering to take part in this focus group. You have been asked to participate as your point of view is important. Your parent has provided us consent for you to take part in this discussion.

Introduction: This focus group discussion is designed to assess your current thoughts and feelings about the school food environment. The focus group discussion will take no more than one hour. May I tape the discussion? (If yes, switch on the recorder).

Anonymity: Despite being taped; I would like to assure you that the discussion will be anonymous. The tapes will be kept safely in a locked facility until they are transcribed word for word; then, they will be destroyed. The transcribed notes of the focus group will contain no information that would allow individual subjects to be linked to specific statements. You should try to answer and comment as accurately and truthfully as possible. I and the other focus group participants would appreciate it if you would refrain from discussing the comments of other group members outside the focus group. If there are any questions or discussions that you do not wish to answer or participate in, you do not have to do so; however please try to answer and be as involved as possible.

Ground rules

- The most important rule is that only one person speaks at a time. There may be a temptation to jump in when someone is talking, but please wait until they have finished.
- There are no right or wrong answers.
- You do not have to speak in any order.
- When you do have something to say, please do so. There are many of you in the group and it is important that I obtain the views of each of you.
- You do not have to agree with the views of other people in the group.
- Does anyone have any questions? (answers).
- OK, let's begin.

Warm-up

- First, I'd like everyone to introduce themselves. Can you tell us your name?

Introductory question

I am just going to give you a couple of minutes to think about the food environment in and around your school.

Guiding questions

Domain: Healthy Foods

Which foods are mainly consumed by people your age? (Probe: examples).

What kind of food do young people your age consider as healthy? (Probe: types and examples of foods. Probe if these foods are easy to obtain?)

What kind of food do young people your age consider as unhealthy? Probe: examples of foods (meals and packaged food), Probe types of cooking methods that are unhealthy

Domain: Practice on nutrition and feeding

Now we're going to think about your eating habits and preferences, both in and outside of school.

What are your favourite foods? (Probe: Do you know what is in it?)

Do you enjoy eating fruits and vegetables? (Probe: Why or why not?)

Do you eat any food when you are at school? If so, what do you eat? (Probe: how frequently do you eat these foods at school? If foods are bought from the tuckshop or informal vendors specify type).

Do you read labels on food products (Probe: Do you understand food labels? What do you look for in a label?).

Do you know how to identify foods high in sugar, salt, and fat? (Probe: Do you know what the daily limit of salt intake is?) (Probe: Have you heard of the sugar-sweetened beverage tax?).

Ultra-processed foods are foods such as polonies, frankfurters, processed cheese etc: Do you eat these foods? (Probe: Reasons. Do you know what is in the contents of these foods?).

Is water available for drinking at school? (Probe: Are you encouraged to drink water? The amount of water drank per day).

Are you able to get the kinds of foods you want to eat? If not, why?

Domain: Health Issues

What health issues are most important to you?

What health issues have you seen among your friends, peer group and family?

Do you think an unhealthy diet (eating foods high in fat, sugar, salt and ultra-processed foods can lead to health issues- if so, describe these health issues associated to an unhealthy diet)?

Domain: Availability

What types of foods and beverages are available in and around your school? (Probe: packaged food, cooked food (method of cooking).

Probe: Which types of foods and beverages would you want to be available in and around school and community? Why?

Domain: NSNP

Do you make use of the NSNP? (If yes or no, provide reasons).

What are your perceptions of the meals provided? (Probe: do you enjoy the meals, is the portion size adequate to relieve hunger,

Do you bring a packed lunch? (Probe: contents of packed lunch, does the school provide any guidelines on the contents of the packed lunch).

Domain: Breakfast

Do you have breakfast at home before coming to school? Probe If no, why and check to see if this is the same practice by most learners attending school.

How would you feel if a breakfast programme was introduced by the school like that of the NSNP lunch programme.

Domain: Vendors

Do you make use of vendors (tuckshops) inside the school and vendors outside the school?

What do young people like you consider when deciding on a place to source food from? Probe: What types of foods do you purchase from formal and informal vendors?
(Probe: price and frequency).

Domain: Marketing and regulations

What type of marketing related to the food industry have you been exposed to in and around school? (Probe: location and types of marketing).

Domain: Challenges in accessing healthy food

What type of foods are you unable to purchase? Probe: Provide reasons. Is healthy food affordable?

What makes it easy or difficult to eat healthy food? Probe: Convenience, Accessibility, Desirability, Taste.

Domain: Physical activity

Do you participate in physical activity at school? Probe (Is it part of a subject or during break time? Probe: Resources available/ indigenous games).

Domain: Nutrition education

Have you been exposed to nutrition education in any subject? (Probe: name of subject/s, content of nutrition education, which grade/s).

During the school year, were you taught in any of your classes about the risks related to eating too many foods high in fat, sugar, or salt?

If you wanted to learn about nutrition and healthy eating, where would you go?

Domain: School policy

Let's talk about your school and how that influences your health.

Do you think your school supports you in being healthy? Why or why not?

What services and/or infrastructure are at your school that supports your health or healthy behaviours? (Probe around nutrition and dietary practices, physical activity if needed).

What health and nutrition activities or services exist in your school?

What health and nutrition activities or services would you want to see at your school?

Domain: Influences

I want to know more about the influence of the people around you on your health and the food you eat.

Outside your school, where do you receive information about health? (Probe: what kind of information do you receive and in what form?)

Outside your school, where do you receive information about eating habits and diet? (Probe: what kind of information do you receive and in what form)

Do you think the people around you influence what you eat? Why or why not? In what ways?

Is your food decision purchase influenced by media and marketing?

Domain: Practice on WASH

How do you get water for your various uses while at school? (Probe: availability of clean and safe drinking water and reliability of its availability across a year; practice of hand washing after activities such as before and after eating, when coming from toilets or when engaged in a physical activity).

I would also like to know about

b) how waste disposal is practised in your school (probe how they participate in waste disposal practices.

How do you ensure your own hygiene and that of others while at school? (Probe to learn their understanding of personal hygiene, what entails in personal hygiene and what do they expect the school to provide for them to exercise hygienic practices)

To what extent is your school safe from vectors and vermin?

How do you perceive the landscaping, gardening, and the overall environment at your school? (Probe also if there are features at school which pose a risk for accidents and injury, Probe: Food gardens: Participation in this activity. What happens to the food?)

Domain: Road safety

Have you received any education on road safety (Probe: when and by whom).

Do you have any challenges with regard to road safety when attending school?

Concluding question

- Of all the things we've discussed today, is there anything else you want to say about your experiences related to your school, health, and nutrition?

Conclusion

Thank you for participating. This has been a very successful discussion.

- Your opinions will be an asset to the study
- We hope you have found the discussion interesting
- If there is anything you are unhappy with or wish to complain about, please contact the local PI or speak to me later
- I would like to remind you that any comments featuring in this report will be anonymous
- Before you leave, please hand in your completed personal details questionnaire

[Stop the recorder]
